# A comparative study on the influence of an ivy preparation and an ivy/thyme combination on the β_2_-adrenergic signal transduction

**DOI:** 10.1016/j.heliyon.2020.e03960

**Published:** 2020-05-15

**Authors:** Hendrik Bussmann, Janka Schulte-Michels, Mara Bingel, Fabio Meurer, Stefan Aatz, Felix Häberlein, Sebastian Franken, Hanns Häberlein

**Affiliations:** Institute of Biochemistry and Molecular Biology, Medical Faculty, University of Bonn, Germany

**Keywords:** Pharmaceutical science toxicology, Biochemistry, Medicine, ivy, thyme, β_2_-adrenergic receptor, Signal transduction, Fluorescence correlation spectroscopy, Single particle tracking, Optics, Dose-response relationship, Drug binding, Natural product, Spectroscopy

## Abstract

The β_2_-adrenergic receptor (β_2_AR) is relevant for surfactant formation in alveolar type 2 cells and reduction of intracellular calcium concentration in bronchial muscle cells and thus for secretolytic and bronchospasmolytic effects. Herbal medicinal products that affect the β_2_AR system are used to treat common cold and bronchitis accompanied with mucus covered and narrowed airways. The present work compares the influence of an ivy preparation and an ivy/thyme combination on the β_2_-adrenergic signal transduction. For receptor binding studies and characterization of the lateral mobility of β_2_AR we have used single molecule detection by fluorescence correlation spectroscopy and single particle tracking. For the determination of both the second messenger cAMP and the internalization of β_2_AR we have generated luciferase based reporter cell lines, which produce a cAMP-dependent luciferase in the cytosol and express β_2_AR with extracellular luciferase moiety in the plasma membrane. While both preparations increased the β_2_AR binding, a significant increase of the cAMP level was observed only for the ivy preparation, which can be explained by the inhibited internalization of HiBiT-tagged β_2_AR under stimulating conditions. In contrast, isoprenaline-mediated internalization of HiBiT-tagged β_2_AR of ivy/thyme combination pre-treated cells was not inhibited. Cells comparatively pre-treated with a thyme preparation did not show inhibition of ß_2_AR internalization either. Furthermore, SNAP-tagged β_2_AR of ivy preparation pre-treated cells, which were not internalized after isoprenaline stimulation, showed a redistribution from fast-to-slowly diffusing β_2_AR. A corresponding redistribution of these receptors was not observed after pre-treatment with both the ivy/thyme combination and the thyme preparation. Comparable to the ivy/thyme combination, no decrease in the intratrack transitioning probability ratio (p23/p32) for fast and slow diffusing β_2_AR was found for the thyme preparation, which, however, significantly decreased for control cells and for pre-treatment with the ivy preparation under stimulating conditions. It can therefore be concluded that the thyme fluid extract fraction in the ivy/thyme combination may have in part a negative effect on the β_2_-adrenergic signal transduction.

## 1 Introduction

Main symptoms of a common cold or bronchitis are narrowed and mucus covered airways. To counteract these symptoms, the human body amplifies adrenaline-mediated activation of β_2_-adrenergic receptors (β_2_AR) of the airways. Elevated β_2_-adrenergic signal transduction enhances cAMP levels in alveolar type 2 cells leading to an increased formation of surfactant, which is secreted onto the cell surface to lower the viscosity of sticky mucus [1]. In bronchial muscle cells increased cAMP levels are also formed under β_2_-adrenergic stimulating conditions, which leads to protein kinase A mediated phosphorylation of myosin light-chain kinase (MLCK) and subsequently prevents the formation of the MLCK-calmodulin/Ca^2+^ complex required for muscle contraction [2]. Additionally, intracellular Ca^2+^ level decreases due to inhibition of Ca^2+^ release from intracellular stores and reduction of membrane Ca^2+^ entry [3]. Through these mechanisms the human body is able to develop secretolytic and bronchospasmolytic activities to overcome narrowed and mucus covered airways.

Extract preparations of common ivy (*Hedera helix* L.) are widely used for the treatment of respiratory tract diseases associated with coughing [4, 5, 6]. Ivy for medicinal use is mainly obtained from eastern european countries. α-Hederin, one of the saponins identified in ivy leaves dried extracts, indirectly inhibits the GRK2-mediated phosphorylation of β_2_AR in human airways smooth muscle (HASM) cells [7]. Since this phosphorylation process is required for receptor internalization, the β_2_AR density on the cell surface of α-hederin treated cells is still high even under stimulation conditions. As a result, the elevated β_2_-adrenergic responsiveness of α-hederin pre-treated HASM and alveolar type 2 (A549) cells was confirmed by increased β_2_AR binding and enhanced cAMP-levels [8]. In addition to pure ivy leaves dried extract preparations, fixed fluid extract combinations of thyme herb and ivy leaves are used for the treatment of acute bronchitis and common cold with concomitant cough and viscous mucus [9, 10]. For a thyme extract, a comparatively weak direct interaction with β_2_AR was described. In addition, an indirect modulatory effect on β_2_AR was found, but the molecular mechanism was not further clarified [11]. Therefore, it can be assumed that α-hederin mediated inhibition of receptor internalization leads to an increased number of β_2_AR, which can be activated and modulated by ingredients of the thyme fluid extract. Thus, the β_2_-adrenergic signaling of an ivy/thyme combination could be superior to a pure ivy preparation. Influences of an ivy preparation and an ivy/thyme combination on the β_2_AR binding and the formation of the corresponding second messenger cAMP using the same cell system have never been compared directly.

In this work, we investigated the influence of a preparation containing an ethanolic (30% [w/w]) ivy leaves dried extract (DER 5–7.5:1) on the binding behaviour of β_2_AR using fluorescence correlation spectroscopy (FCS) and on the intracellular cAMP-level as readout parameter of β_2_AR activation. Furthermore, the influence of the ivy preparation on the isoprenaline-induced β_2_AR internalization and regulation of the lateral mobility of non-internalized β_2_AR was examined by means of single particle tracking (SPT). In parallel, we analysed a fixed fluid extract combination of thyme herb and ivy leaves (fluid extract of thyme herb: 1:2–2.5, ammonia solution 10% [w/w]:glycerol 85% [w/w]:ethanol 90% [v/v]:water [1:20:70:109]; fluid extract of ivy leaves: 1:1, ethanol 70% [v/v]) and a thyme preparation containing a fluid extract of thyme herb similarly prepared to the ivy/thyme combination. Aim of the work was to preclinically investigate whether a co-medication of ivy with other plant extracts can increase the therapeutic effects of ivy or not. Therefore a combination of ivy and thyme was used to study these effets. Since a corresponding comparative clinical study is not yet available, we investigated this question by means of cell-based experiments.

## 2 Results

### 2.1 Determination of α-hederin

**Figure 1:**
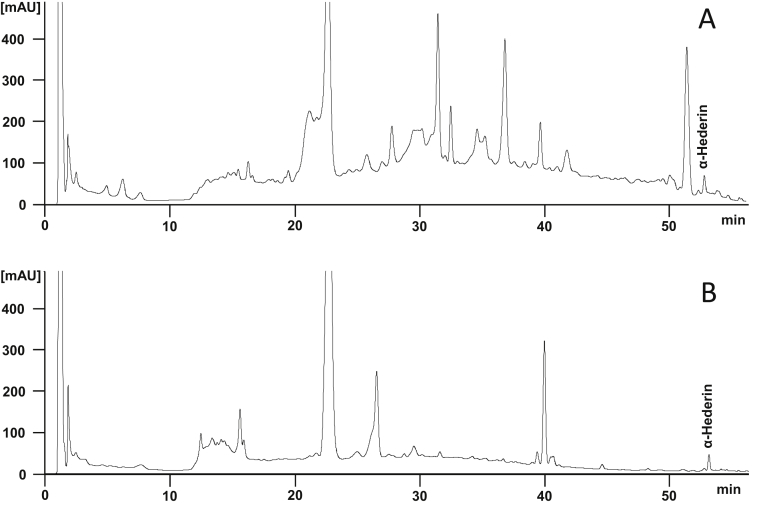
HPLC fingerprint analysis of ivy/thyme combination (A) und ivy preparation (B).

Initially, the content of α-hederin was determined by HPLC with the result of 7.7 mg/100 ml for the ivy preparation and 8.8 mg/100 ml for the ivy/thyme combination (Figure 1).

### 2.2 β_2_AR binding studies

**Figure 2:**
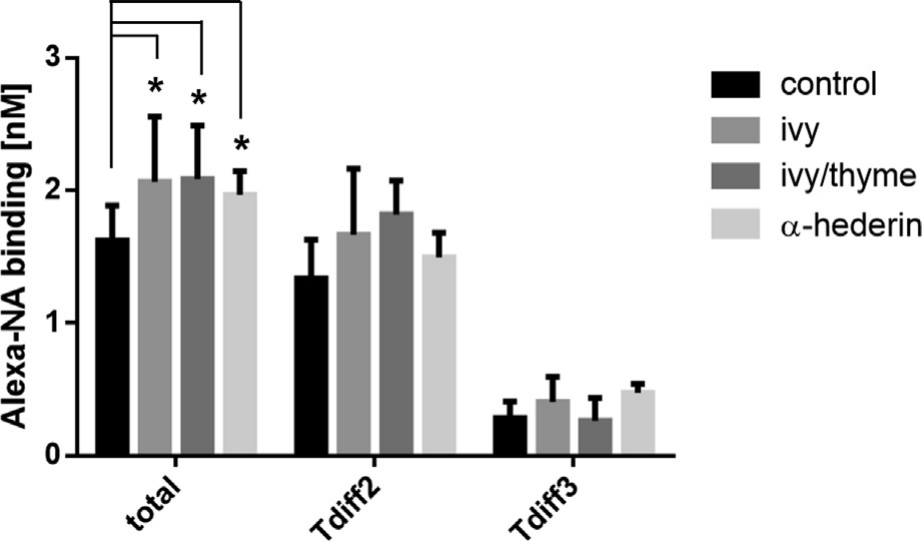
Influence of ivy preparation and ivy/thyme combination on the β_2_AR binding of A549 cells, compared to α-hederin pretreated cells and non-treated control cells. (n ≥ 24 from at least 4 independent experiments, ∗p < 0.05).

The binding behaviour of β_2_AR and the diffusion of β_2_AR-ligand complexes on the surface of A549 cells were investigated using FCS and Alexa-NA (norepinephrine coupled to the fluorescent dye Alexa532) as β_2_AR agonist. A549 cells pre-incubated with 5 nM Alexa-NA for 15 min revealed a total binding of 1.62 ± 0.27 nM (32.4 ± 10.2%), distributed in receptor-ligand complexes (RLC) with different diffusion time constants of τdiff2 = 0.32 ± 0.10 ms (1.33 ± 0.29 nM) for free diffusing RLC in the plasma membrane and τdiff3 = 23.90 ± 16,70 ms (0.29 ± 0.12 nM) for RLC with hindered lateral diffusion behaviour (n = 70) (Figure 2). Diffusion time constant of unbound Alexa-NA in solution was τdiff1 = 0.047 ± 0.006 ms.

A549 cells pre-treated for 24 h with both the ivy preparation and the ivy/thyme combination each in a concentration corresponding to 1 μM α-hederin revealed changes in Alexa-NA binding (Figure 2). Pre-incubation with the ivy preparation significantly increased the level of Alexa-NA binding from 1.62 ± 0.27 nM (n = 70) to 2.07 ± 0.49 nM (n = 98, p < 0.05) compared to control cells. Pre-incubation with the ivy/thyme combination significantly increased the ligand binding to 2.09 ± 0.40 nM (n = 24, p < 0.05). A similar effect was found for α-hederin pre-treated cells. One μM of α-hederin served as positive control and increased the Alexa-NA binding to 1.97 ± 0.18 nM (n = 67, p < 0.05). Notably, the increase in Alexa-NA binding for ivy pre-treated cells was found for both receptor-ligand mobilities, whereas for ivy/thyme pre-treated cells, the increased Alexa-NA binding was selectively observed for free diffusing RLC with τdiff 2 (Figure 2, Table 1). The τdiff values were not influenced by the test substances (Table 1).

**Table 1:** Influence of ivy preparation, ivy/thyme combination, and α-hederin on β_2_AR binding and the presence of receptor-ligand complexes with different lateral mobilities on A549 cells. (n ≥ 24 from at least 4 independent experiments, ∗p < 0.05).

	Alexa-NA binding			Diffusion time constants of β_2_-adrenergic receptor-Alexa-NA complexes	
	total [nM]	τdiff2 [nM]	τdiff3 [nM]	τdiff2 [ms]	τdiff3 [ms]
control	1.62 ± 0.51	1.33 ± 0.29	0.29 ± 0.12	0.32 ± 0.10	23.9 ± 16.7
α-hederin	1.97 ± 0.18∗	1.49 ± 0.19	0.48 ± 0.07	0.47 ± 0.21	47.6 ± 68.1
ivy	2.07 ± 0.49∗	1.66 ± 0.50	0.41 ± 0.14	0.35 ± 0.41	62.1 ± 48.5
ivy/thyme	2.09 ± 0.40∗	1.82 ± 0.25	0.27 ± 0.17	0.62 ± 0.19	33.5 ± 19.9

### 2.3 Determination of cAMP levels

**Figure 3:**
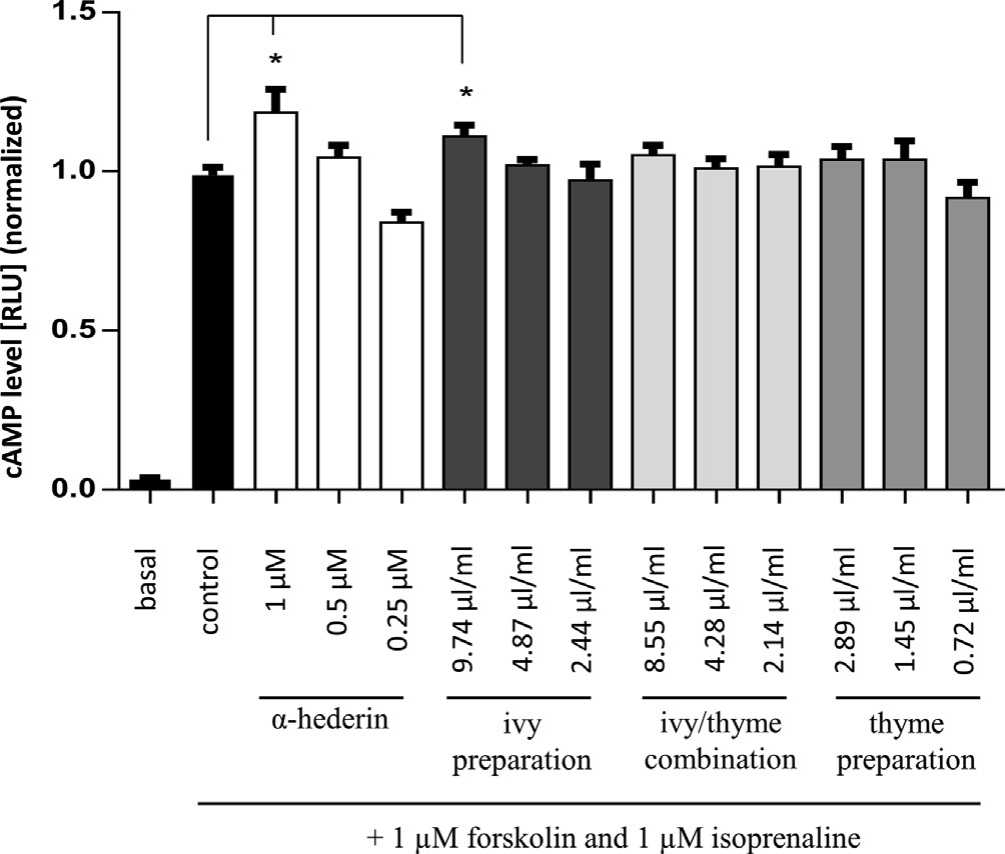
Influence of ivy preparation, ivy/thyme combination, and thyme preparation on the cAMP level in HEK293 GloSensor™ cells after stimulation with forskolin and isoprenaline. Amounts of ivy preparation and ivy/thyme combination corresponding to 0.25 μM, 0.5 μM, and 1 μM α-hederin in the assay were used. For the thyme preparation amounts were used corresponding to fluid extract of thyme herb present in the ivy/thyme combination. (n = 12 from 3 independent experiments, ∗p < 0.05).

In order to analyse the influence of the altered β_2_AR-ligand binding on the intracellular cAMP level, HEK293 cells expressing a luciferase fused with a cAMP binding domain (HEK293 GloSensor™ cells) were investigated under stimulating conditions using 1 μM forskolin and 1 μM isoprenaline for 20 min. Compared to stimulated control cells, pre-incubation with the ivy preparation corresponding to 1 μM α-hederin (see 5.10) for 16 h was found to increase the intracellular cAMP level significantly from 0.99 ± 0.02 to 1.11 ± 0.04 (n = 10) (Figure 3). This effect was dose dependent. A comparable effect with a relative increase of the cAMP level to 1.18 ± 0.07 was observed for cells pre-treated with 1 μM α-hederin. In contrast, for cells pre-incubated with ivy/thyme combination in a concentration which corresponded to 1 μM α-hederin, a slightly increased cAMP level to 1.05 ± 0.07 was observed which was not statistically significant (Figure 3). Pre-incubation with 2.89 μl/ml thyme preparation, which corresponded to the amount of thyme fluid extract present in the ivy/thyme combination, also slightly increased the cAMP level to 1.04 ± 0.07 compared to control (Figure 3).

### 2.4 Isoprenaline mediated internalization of HiBiT-tagged β_2_AR

**Figure 4:**
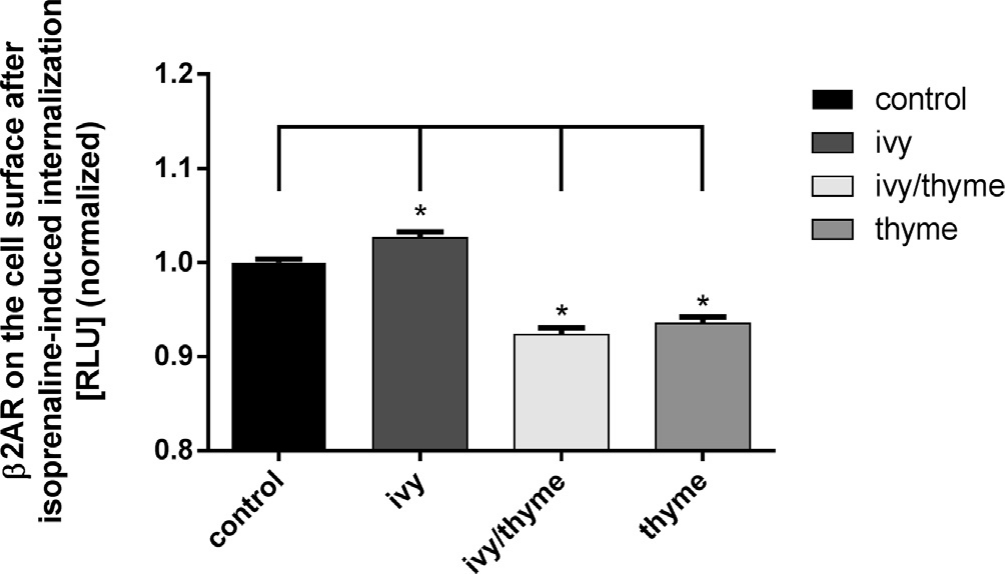
Remaining β_2_AR on the surface of HEK293-HiBiT-β_2_AR cells after isoprenaline induced internalization. Amounts of 9.74 μl/ml of ivy preparation and 8.55 μl/ml of ivy/thyme combination corresponding to 1 μM α-hederin in the assay were used. 2.89 μl/ml thyme preparation was used corresponding to fluid extract of thyme herb present in the ivy/thyme combination. (n = 100 from 2 independent experiments, ∗p < 0.05).

Internalization of β_2_AR was investigated on HEK293-HiBiT-β_2_AR cells expressing HiBiT-tagged β_2_AR after stimulation with 5 μM isoprenaline for 30 min. Compared to stimulated control cells, 2.6% more β_2_AR were found on the cell surface after pre-treatment with ivy preparation. In contrast, the number of β_2_AR decreased by 7.7% and 6.5% after pre-treatment with ivy/thyme combination and thyme preparation, respectively (Figure 4). Thus, only the ivy preparation inhibited the internalization of β_2_AR under stimulating conditions, whereas an increased internalization was observed for the other two preparations.

### 2.5 Lateral diffusion behaviour of SNAP-tagged β_2_AR

**Figure 5:**
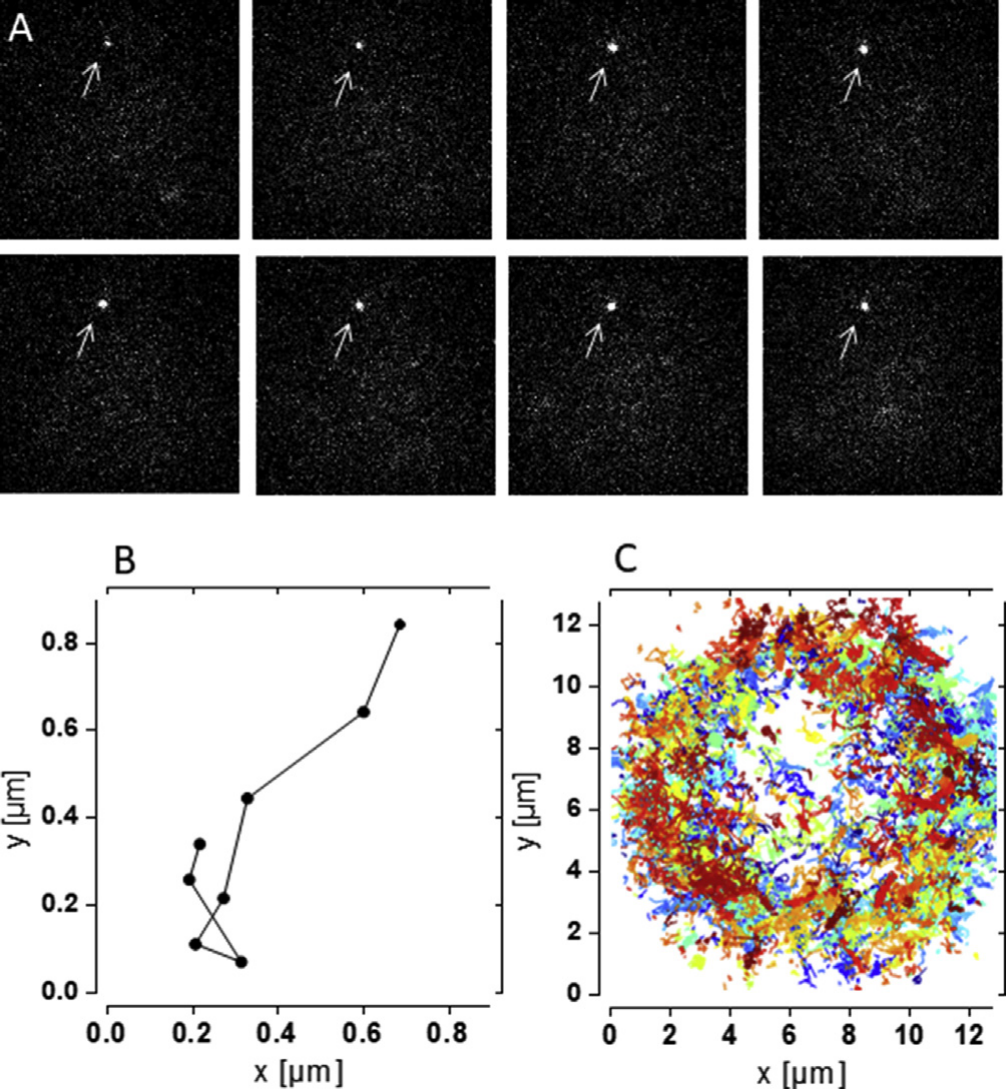
SPT of SNAP-tagged β_2_AR overexpressed in HEK293 cells and coupled to BG-CF640R. Individual frames (A), generation of a single trajectory (B), and overlay of all trajectories (C) from a single SPT recording acquired at 20 frames per second of a non-treated control cell for 50 s.

Lateral diffusion behaviour of SNAP-tagged β_2_AR overexpressed in HEK293 (HEK293-β_2_AR-SNAP) cells was investigated using SPT. In order to monitor trajectories of β_2_AR, the SNAP-tag was coupled to BG-CF640R (Figure 5).

**Figure 6:**
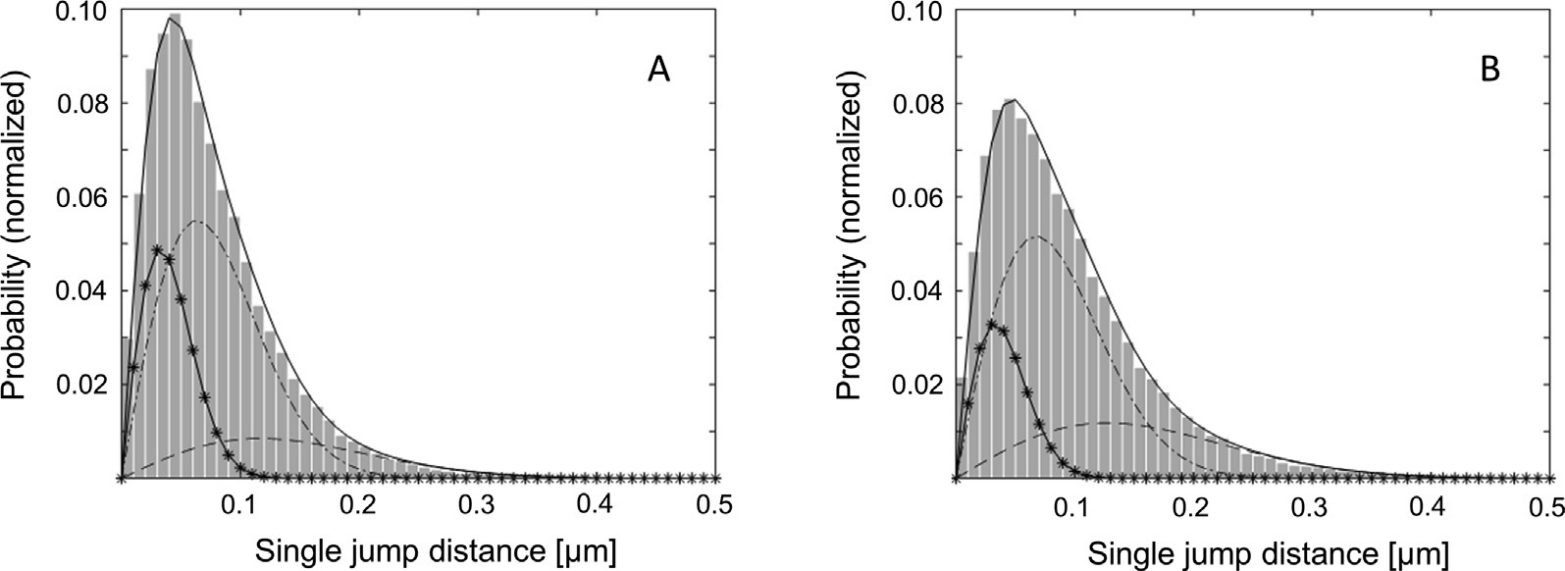
Jump distance distribution of non-treated HEK293-β_2_AR-SNAP cells before (A) and after (B) isoprenaline stimulation. Presence of different diffusion coefficients are separately displayed (D1 (^_._._^), D2 (-_∗_-_∗_-), D3 (----)).

Jump distances of all trajectories were recorded in order to create a jump distance distribution, from which three different diffusion coefficients were determined. For untreated HEK293-β_2_AR-SNAP control cells diffusion coefficients were observed with D1 = 0.011 μm^2^/s (17%) and D2 = 0.046 μm^2^/s (57%) for almost immobile (diffusion state S1) and slow diffusing (diffusion state S2) β_2_AR, respectively, while D3 = 0.162 μm^2^/s (26%) was found for fast diffusing (diffusion state S3) β_2_AR (n = 20) (Figure 6, Table 2).

**Table 2:** Different diffusion coefficients and intratrack transitioning probability ratios of SNAP-tagged β_2_AR overexpressed in HEK293 cells. n = 20 from 2 independent experimnets, ∗p < 0.05.

	D1		D2		D3		ratio
	μm^2^/s	%	μm^2^/s	%	μm^2^/s	%	p23/p32
control cells	0.011	17	0.046	57	0.162	26	0.41
control cells + isoprenaline	0.011	27∗	0.040	57	0.135	16∗	0.25
cells pre-treated with ivy preparation	0.010	14	0.044	52	0.151	33	0.56
cells pre-treated with ivy preparation + isoprenaline	0.008	25∗	0.029	52	0.110	23∗	0.38
cells pre-treated with ivy/thyme combination	0.011	20	0.011	55	0.135	25	0.42
cells pre-treated with ivy/thyme combination + isoprenaline	0.008	19	0.027	54	0.086	27	0.43
cells pre-treated with thyme preparation	0.019	11	0.054	54	0.150	35	0.41
cells pre-treated thyme preparation + isoprenaline	0.021	13	0.052	55	0.156	32	0.45

**Figure 7:**
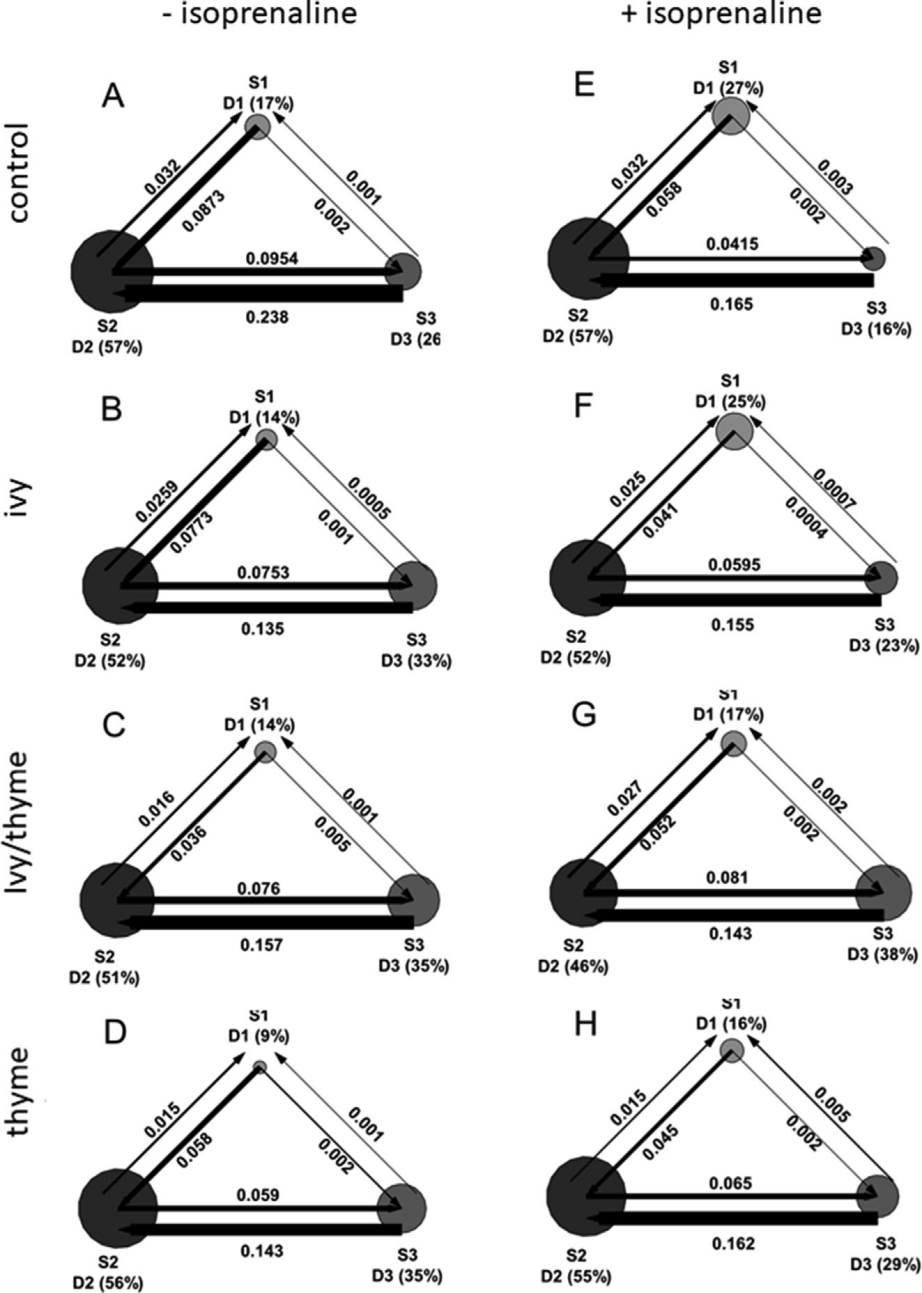
Trajectories of HEK293-β_2_AR-SNAP cells pre-treated with ivy preparation, ivy/thyme combination or thyme preparation in comparison to control cells before (A–D) and after isoprenaline stimulation (E–G) were analysed by vbSPT. Circle area and arrow thickness are proportional to occupancy of diffusion coefficients (D1-D3) and intratrack transitioning propability between S1, S2, and S3. (n = 20 from 2 independent experiments).

To further analyse and quantify the different diffusion states, a variational Bayes analysis (vbSPT) [14] was applied. Transition probabilities indicated, that β_2_AR frequently switched diffusion states in sequential order from S1 to S3 and backwards. Switches between S1⇄S2 and S1⇄S3 were relatively rare, while the transition from S3→S2 clearly dominated (Figure 7).

For non-treated control cells the intratrack transitioning probability for β_2_AR to change the diffusion state from S2 to S3 (p23) or vice versa from S3 to S2 (p32) can be described by the ratio p23/p32 = 0.41. Stimulation with 10 μM isoprenaline for 30 min induced internalization of β_2_AR, reducing the number of trajectories to 78 per 1000 frames per cell compared to 263 trajectories per 1000 frames per cell in control cells. For the β_2_AR remaining on the cell surface, stimulation did not affect the size of the diffusion coefficients, but significantly changed their proportions with D1 = 27%, D2 = 57%, and D3 = 16% (Table 2). Additionally, intratrack transitioning probability ratio of p23/p32 decreased to 0.25. A comparable redistribution of β_2_AR was observed after pre-treatment with ivy preparation corresponding to 1 μM α-hederin for 12 h followed by isoprenaline stimulation. Occurrence significantly changed for D1 from 14% to 25% and for D3 from 33% to 23% whereas D2 stayed at 52% compared to non-stimulating conditions. Accordingly, cells pre-treated with ivy preparation showed an intratrack transitioning probability ratio of p23/p32 = 0.56 which significantly decreased to 0.38 after isoprenaline stimulation (Table 2). In contrast, pre-treatment with ivy/thyme combination corresponding to 1 μM α-hederin and subsequent stimulation with isoprenaline did not alter the occurrence of D1 with 19%, D2 with 54%, and D3 with 27% compared to non-stimulating conditions. Accordingly, the intratrack transitioning probability ratio did not change (Table 2). The thyme fluid extract, as part of the ivy/thyme combination, could be responsible for these effects. To test this assumption, a thyme preparation was investigated that according to the manufacturer contains a comparably prepared thyme fluid extract as the ivy/thyme combination. The amount of 2.89 μl/ml used for the pre-treatment of HEK293-β_2_AR-SNAP cells corresponded to the amount of thyme fluid extract present in the tested ivy/thyme combination. Evaluation of vbSPT data before and after isoprenaline stimulation revealed no significant redistribution of β_2_AR with D1 from 11% to 13%, D2 from 54% to 55%, and D3 from 35% to 32%. Furthermore, intratrack transitioning probability ratio of p23/p32 from 0.41 to 0.45 did not change under stimulating conditions (Figure 7, Table 2). Obviously, the isoprenaline-induced regulation of the lateral mobility of β_2_AR is negatively influenced in HEK293-β_2_AR-SNAP cells pre-treated with the thyme preparation.

## 3 Discussion

Physiological mechanisms targeting secretolytic and bronchospasmolytic effects are based on the formation of the second messenger cAMP after stimulation of β_2_AR in the respiratory tract. cAMP is crucial because it induces both the formation of surfactant in alveolar type 2 cells and a decrease of intracellular calcium concentration in bronchial muscle cells [1, 2, 3]. For the treatment of respiratory tract diseases with mucus covered and narrowed airways drugs are useful, which support these physiological mechanisms. This can be achieved by direct activation of β_2_AR by administration of β_2_-agonist [17, 18]. Alternatively, an increase in the β_2_-adrenergic respiratory responsiveness is reasonable in this context. As an active ingredient of therapeutically used ivy leaves dry extracts, α-hederin increases β_2_-adrenergic responsiveness for both alveolar type 2 cells and bronchial muscle cells by indirectly inhibiting GRK2-mediated phosphorylation of activated β_2_AR [7]. As a result, the β_2_AR remains on the cell surface under stimulating conditions and is therefore not internalized, leading to increased β_2_AR binding [8].

This finding was confirmed by own β_2_AR binding studies on A549 cells using an ivy preparation and an ivy/thyme combination, each containing an α-hederin concentration of 1 μM in the assay (Figure 2). For both preparations, a comparable increase in β_2_AR binding was found. Since similar β_2_AR densities under stimulating conditions can be assumed for both preparations, the β_2_-adrenergic signaling could be stronger under the influence of the ivy/thyme combination, because thyme extracts could presumably contain ingredients that activate the β_2_AR to a certain degree agonistically [11]. In our receptor binding studies, we used the fluorescent ligand Alexa-NA, whose β_2_-agonistic profile and binding properties were extensively characterized on different cell types [12]. If thyme ingredients significantly bind to β_2_AR, these substances would have to displace receptor-bound Alexa-NA. However, since the Alexa-NA binding was not lower in the presence of the ivy/thyme combination compared to the ivy preparation, no increased β_2_-adrenergic signaling can be assumed for the ivy/thyme combination. Radioreceptor binding studies on lung membrane suspensions using [125]-iodocyanopindolol as radioligand also showed no significant binding activitiy of the tested thyme extract [11].

HEK293 cells expressing a biosensor with cAMP binding domain fused to a luciferase were used to determine intracellular cAMP concentrations. Upon cAMP binding, a conformational change of the fusion protein is induced, leading to luciferase activation and oxidation of the substrate luciferin to oxyluciferin. This reaction emits chemiluminescent light which correlates with the cAMP concentration. Cells pre-treated with the ivy preparation showed a significant and dose-dependent increased cAMP level under adenylyl cyclase and β_2_AR stimulating conditions as found for α-hederin as a positive control (Figure 3). A similar effect was also observed on HASM cells for α-hederin [8]. Remarkably, the ivy/thyme combination mediated only a tendentially weak increase in cAMP level, although a significant increase in β_2_AR binding was observed in our FCS experiments. To clarify this surprising finding, the cAMP level of cells was examined after pre-treatment with a thyme preparation. Interestingly, the cAMP level was not affected by the thyme preparation compared to control. In contrast an elevated cAMP level was found in human COPD airway epithelial cells pre-treated with a thyme extract under non-stimulating conditions [19]. Although the effect was comparable to isoprenaline, the authors did not comment on a possible mode of action. Comparing the cAMP levels, the thyme fluid extract in the ivy/thyme combination may affect the signaling of isoprenaline-activated β_2_AR and thus their functionality.

The increased cAMP level of cells pre-treated with the ivy preparation can be explained by an inhibited internalization of β_2_AR and thus by an increased β_2_-adrenergic responsiveness. In contrast, β_2_AR internalization was increased by pre-treatment with an ivy/thyme combination compared to control, which may contribute to the cAMP level not being increased as expected. The functionality of membrane-bound β_2_AR can be characterized among others by lateral diffusion behaviour. Terbutaline-activated β_2_AR lose their almost free and fast diffusion behaviour and become increasingly immobile as soon as a certain signal strength is reached [20]. This observation suggests that with increasing signal strength more and more receptors leave their functional microdomains and are redistributed into coated pits. A comparable result has been shown by our investigations. Under stimulating conditions and subsequent β_2_AR internalization, in HEK293-β_2_AR-SNAP cells the fraction of rapidly diffusing β_2_AR with D3 decreased while the fraction of immobile receptors with D1 increased. Furthermore, discrete diffusive states S1, S2, and S3 were identified and the probabilities for β_2_AR to change between S2 and S3 within a single track were calculated (14). Accordingly, the intratrack transitioning probability ratio p23/p32 halved to a value of 0.25, compared to non-stimulated control cells. Thus, HEK293-β_2_AR-SNAP control cells respond to the binding of isoprenaline to β_2_AR and relocate desensitized β_2_AR in coated pits, which explains the large number of immobile receptors with diffusion state S1. Hegener et al. (2004) investigated the lateral mobility of β_2_AR on the surface of different cell types using FCS [12]. Fast lateral mobility of the β_2_AR-Alexa-NA complex was detected immediately after addition of the ligand, whereas hindered mobility was observed after a delay of 15–20 min in A549 cells. The authors conclude that receptor regulation is required to form receptor-ligand complexes with slow lateral mobility. Comparable results were obtained from isoprenaline-stimulated HEK293-β_2_AR-SNAP cells pre-treated with ivy preparation showing a decreasing fraction of fast diffusing β_2_AR with D3 and an increasing fraction of immobile β_2_AR with D1 and a reduction in the intratrack transitioning probability ratio p23/p32 (Figure 7, Table 2). This finding was supported by data from the receptor binding study using FCS. Compared to the control, cells pre-treated with ivy preparation showed an increase in receptor-ligand complexes with impaired lateral mobility (τdiff3) (Figure 2). The ivy preparation has apparently no influence on the isoprenaline-mediated regulation of the lateral mobility of β_2_AR, observed by means of SPT. Since the lateral mobility of β_2_AR usually changes during signal transduction, it can be assumed that the ivy preparation does not inhibit β_2_-adrenergic signaling. Rather, it has been shown that α-hederin increases the β_2_-adrenergic responsiveness of A549 cells and HASM cells and improves isoprenaline-mediated relaxation of methacholine pre-contracted bovine tracheal smooth muscles [8, 21]. These findings have recently been substantiated by the α-hederin-induced inhibition of GRK2-mediated phosphorylation of β_2_AR [7]. HEK293-β_2_AR-SNAP cells pre-treated with ivy/thyme combination showed no changes in the lateral mobility of β_2_AR after stimulation with isoprenaline. Also in the FCS receptor binding study there is no change in the occurrence of receptor-ligand complexes with hindered lateral mobility (τdiff3) after pre-treatment with the ivy/thyme combination (Figure 2). Furthermore, the intratrack transitioning probability ratio p23/p32 was not affected under stimulating conditions (Figure 7, Table 2). It seems as if ingredients of thyme extract interfere with β_2_-adrenergic signaling and subsequently prevent isoprenaline-mediated activation and regulation of lateral mobility of β_2_AR. To answer this question, a thyme preparation was examined containing a fluid extract similar prepared as the fluid extract in the ivy/thyme combination. HEK293-β_2_AR-SNAP cells were pre-treated with a quantity of thyme preparation that corresponded to the amount of thyme fluid extract in the ivy/thyme combination. Remarkably, cells pre-treated with the thyme preparation did not show a significant isoprenaline-induced redistribution of fast-to-slow diffusing β_2_AR. Accordingly, the reduction in intratrack transitioning probability ratio p23/p32 after isoprenaline stimulation found for control cells was not observed for HEK293-β_2_AR-SNAP cells pre-treated with the thyme preparation.

A variety of other plant extracts show stimulatory effects on the ß_2_AR of tracheal smooth muscles [23]. These plants basically have the potential to be effective in the treatment of a cold or bronchitis. However, no approved drugs have been developed from most of these plants. Often there are no clinical data or they are not convincing. Guidelines of medical societies recommend not only mono-preparations of ivy, cineole, myrtol, and *Pelargonium sidoides*, but also combination preparations of ivy and thyme as well as primrose and thyme for the treatment of cough, because here the effectiveness has been proven in randomized clinical studies [24]. A combination preparation should be superior to the mono-preparation with regard to the clinical effect. This has not yet been shown for ivy combination preparations. However, comparative clinical studies are important to justify the use of combination products and to ensure optimal therapy. Preclinical studies can never replace clinical studies, but they can be used as a basis for decisions on the use of a drug.

## 4 Conclusion

HEK293-β_2_AR-SNAP cells pre-treated with an ivy preparation showed an increased β_2_AR binding and enhanced cAMP levels under stimulating conditions. Following β_2_-agonist binding, β_2_AR usually become partly redistributed from functional microdomains to coated pits where they display a immobile lateral diffusion behaviour. This regulatory process was not influenced by the ivy preparation. The hypothesized synergistic activation of β_2_-adrenergic signaling by an ivy/thyme combination [22] could not be confirmed by β_2_AR binding studies, determination of the second messenger cAMP and characterization of diffusion states of β_2_AR still present on the cell surface after isoprenaline-induced internalization. Although an elevated β_2_AR binding was found for the ivy/thyme combination, the expected increase in cAMP formation was not clearly observed. Remarkably, HEK293-β_2_AR-SNAP cells pre-treated with an ivy/thyme combination showed no change in β_2_AR fractions with different diffusion states under stimulating conditions. Comparable findings were observed after pre-treatment with the thyme preparation. It can therefore be assumed that ingredients of thyme fluid extracts in part abolish the ivy extract mediated increase in β_2_-adrenergic signaling of HEK293-β_2_AR-SNAP cells.

## 5 Material and methods

### 5.1 Substances

α-Hederin was obtained from HWI pharma services GmbH, Rülzheim, Germany. Isoprenaline and forskolin were delivered by Sigma-Aldrich, Crailsheim, Germany. cAMP response plasmid “-22F cAMP” GloSensor™ was obtained from Promega GmbH, Mannheim, Germany. Nano-Glo® HiBiT Extracellular Detection System was obtained from Promega. All other reagents were purchased from Merck, Darmstadt, Germany, if not otherwise stated. Thyme preparation containing 496.7 mg fluid extract of thyme herb (1:2–2.5, ammonia solution 10% [w/w]:glycerol 85% [w/w]:ethanol 90% [v/v]:water [1:20:70:109]) per ml, ivy preparation containing 700 mg ivy leaves dried extract (DER 5–7.5:1, 30% ethanol) per 100 ml and fixed fluid extract combination of thyme and ivy leaves (16.8 g fluid extract of thyme herb: 1:2–2.5, ammonia solution 10% [w/w]:glycerol 85% [w/w]:ethanol 90% [v/v]:water [1:20:70:109] per 100 ml; 1.68 g fluid extract of ivy leaves: 1:1, ethanol 70% [v/v] per 100 ml) were purchased from local pharmacy store.

### 5.2 Alexa532-norepinephrine

Synthesis, identity and binding behaviour of Alexa532-norepinephrine (Alexa-NA) at β_2_AR were reported by our group previously [12].

### 5.3 BG-CF640R

Synthesis and identity of BG-CF640R were reported by Bosch et al. (2014) [13].

### 5.4 HEK293 cells overexpressing SNAP-tagged β_2_AR

Human embryonic kidney cells (HEK293) obtained from DSMZ (German Collection of Microorganisms and Cell Culture, Braunschweig, Germany) were cultivated in DMEM medium supplemented with 2 mM L-glutamine, 100 U/mL penicillin, 100 μg/mL streptomycin, and 10% fetal calf serum. HEK293 cells were transfected with a commercially available plasmid coding for β_2_AR with N-terminal SNAPtag (New England Biolabs, Frankfurt, Germany, catalog number #N9184). Transfection was done by calcium phosphate transfection method. Cells were seeded in 12 well plates and allowed to attach for at least 24 h. 6.5 μl of 2 M CaCl_2_ and 50 μl sterile water were mixed with 1 μg plasmid DNA. The mixture was added dropwise to 2x HBS buffer (pH 7.13, 42 mM HEPES, 274 mM NaCl, 10 mM KCl, 1.4 mM Na_2_HPO_4_x 2H_2_0, 15 mM Glucose). After 30 min the mixture was added to the cells. The medium was changed to fresh DMEM on the next day containing 750 mg/mL G418 for selection. Individual clones were selected in cloning rings and seeded in distinct wells of a 12 well plate. The clone with best β_2_AR-SNAP expression was used.

### 5.5 HEK293 cells expressing a sensor for cAMP

HEK293 cells were transfected by Amaxa electroporation technology using Nucleofector® II. For each transfection 2 × 10^6^ HEK293 cells were diluted in 100 μl of a self-made electroporation buffer (5 mM KCl, 15 mM MgCl_2_, 15 mM HEPES, 50 mM NaCl, 150 mM, Na_2_HPO_4_/NaH_2_PO_4_, pH 7.2) with 2 μg GloSensor™ cAMP Plasmid DNA and transfected with the program Q-001. Afterwards they were transferred to a 10 cm plate with 10 ml fresh, fully supplemented DMEM medium. After 24 h the medium was changed to fully supplemented DMEM medium containing 100 μg/ml hygromycin for selection. When they were grown to confluency cells were diluted into a 96 well plate to generate a single cell dilution. Grown colonies were tested for functionality with the assay procedure described below.

### 5.6 HEK293 cells overexpressing HiBiT-tagged β_2_AR

Internalization of the β_2_AR was performed using the Nano-Glo® HiBiT Extracellular Detection System from Promega. For this purpose, the human β_2_AR was provided with an N-terminal 11 amino acids long tag (HiBiT, VSGWRLFKKIS) using PCR technology. HEK293 cells were transfected with the HiBiT-β_2_AR DNA construct by polyethyleneimine (PEI) method. Briefly, cells were seeded in 12 well plates and allowed to grow until a confluency of ~80 % was reached. Before transfection medium was changed to 900 μl fresh medium. Two μg DNA were diluted in 100 μl 150 mM NaCl. 6.6 μl of a PEI stock solution (10 mg PEI in 10 ml ddH2O) were added. After an incubation of 10 min at room temperature, 100 μl of the mixture was added to the cells. Culture vessel was centrifuged for 5 min at 280 g. After 24 h, medium was changed to fresh DMEM containing antibiotic for selection of HEK-HiBiT-β_2_AR positive cell clones (150 μg/ml zeocin).

### 5.7 Cell Culture

The human lung carcinoma cell line A549 obtained from DSMZ was cultivated in RPMI 1640 medium supplemented with 2 mM L-glutamine, 100 U/mL penicillin, 100 μg/mL streptomycin, and 10 % fetal calf serum (all Gibco by Life Technologies, Darmstadt, Germany). For FCS experiments cells were seeded in a density of 1.7–3.4 × 10^4^ cells/cm^2^ on heat-sterilized glass coverslips (#1, ∅18 mm, VWR International GmbH, Langenfeld, Germany) and maintained in 12x multi-well chambers (Nunc, Langenselbold, Germany) at 37 °C and 5% CO_2_. Cells were used for FCS experiments after three to four days in culture at 80–90 % confluency.

Human embryonic kidney cells (HEK293) obtained from DSMZ (No. ACC 305) were cultivated in DMEM medium (Life Technologies, Carlsbad, CA) supplemented with 100 units/ml penicillin, 100 μg/ml streptomycin, and 10% fetal calf serum. Cells were maintained by five-to ten-fold dilutions with fresh medium every 3–4 days.

For SPT measurements, HEK293 cells overexpressing SNAP-tagged β_2_AR (HEK293-β_2_AR-SNAP) were grown on PDL coated 18 mm glass cover slips in 12-well plates. Cells were cultivated at 37 °C, 5% CO_2_ in DMEM medium (Gibco 31885-023) containing 10% fetal calf serum, 100 units/ml penicillin and 10 μg/ml streptomycin and used for SPT experiments at 80–90% confluency.

### 5.8 High-performance liquid chromatography

The following instrumentation was used: HPLC pump MSDS 600 E, diode array detector 996, WISP 712 and MILLENIUM V2.1 software were form Waters (Eschborn, Germany). For separation a LiChrospher® 100 RP18 column (125 mm × 4 mm, 5 μm, Merck, Darmstadt, Germany) and the eluents A (distilled water/acetonitrile = 88/4 (v/v), pH = 2 (H_3_PO_4_)) and B (acetonitrile) were used for the following linear gradient: 0–9 min 0% B (isocratic), 9–10 min to 6% B, 10–25 min to 15% B, 25–50 min to 40% B, 50–55 min to 60% B, and 55–65 min to 100% B. Flow rate was 1 ml/min. The ivy preparation and the ivy/thyme combination were diluted with methanol in a volume ratio of 1:2. 60 μl of these solutions were injected. 7.8 mg α-hederin reference substance were dissolved in 25.00 ml methanol, of which 40 μl were injected. Assignment of α-hederin in the HPLC chromatogram was performed through comparison of UV data and retention time of the corresponding reference substance.

### 5.9 Fluorescence correlation spectroscopy (FCS)

FCS measurements were performed with a ConfoCor 1® instrument (Zeiss, Jena, Germany). For excitation the 514 nm line of an argon laser (LGK 7812 ML 2, Lasos, Jena, Germany) was separated by an appropriated filter (515 FS 10–25, Andover, Salem, USA) and focussed through a water immersion objective (C-Apochromat, 63x, NA 1.2) into the sample (laser power: *p*_514nm_ = 2.4 kW/cm^2^). In order to separate the fluorescence light, a dichroic filter (FT540, Andover) and a bandpass filter (EF530-600, Andover) were integrated into the emission light path. After passing a variable pinhole (40 μm), the intensity fluctuations were detected by an avalanche single photon counting module (SPCM-AG Series, PerkinElmer Optoelectronics, Fremont, Canada). Using the MATLAB Software (version R2009a, The MathWorks Inc., Natrick, MA) autocorrelation curves were evaluated with the autocorrelation function (equation 1):

(1) $$G\left(\tau \right)=1+\frac{\sum_{j=1}^{n}Q_{j}^{2}N_{j}}{{\left[\sum_{j=1}^{n}Q_{j}N_{j}\right]}^{2}}\frac{1}{1+\tau /\tau_{D_{j}}}\sqrt{\frac{1}{1+{\left(\omega_{0}/z_{0}\right)}^{2}{\tau /\tau_{D}}_{j}}}$$

with

(2) $$\tau_{D_{j}}=\frac{\omega_{0}^{2}}{4D_{j}}$$

and

(3) $$Q_{j}=\sigma_{j}\eta_{j}g_{j}$$

where *N*_*j*_ is the average number of molecules of the species *j* in the volume element, τ_D*j*_ is the diffusion time constant of the species *j*, τ is the correlation time, *ω*_0_ is the radius of the observation volume in the focal plane, *z*_0_ is the radius of the observation volume in the z-direction, *D*_*j*_ is the translational diffusion coefficient of the species *j*, *Q*_*j*_ is the quantum yield factor, *σ*_*j*_ is the absorption coefficient, *η*_*j*_ is the fluorescence quantum yield, *g*_*j*_ is the fluorescence detection efficiency of the species *j*.

### 5.10 Receptor binding studies

A549 cells were pre-incubated for 24 h by adding 9.74 μl/ml of the ivy preparation (corresponds to 1 μM α-hederin), 8.55 μl/ml of the ivy/thyme combination (corresponds to 1 μM α-hederin) or 1 μM α-hederin to the culture medium. A maximum of 0.1 % ethanol was added as vehicle to control cells and treated cells. Prior to measurements cells were washed three times with Locke's solution pH 7.3 (154.0 mM NaCl, 5.6 mM KCl, 2.3 mM CaCl_2_ dihydrate, 1.0 mM MgCl_2_ hexahydrate, 3.6 mM NaHCO_3_, 5.0 mM HEPES, 2.0 mM D-(+)-glucose monohydrate). The coverslips were mounted on a coverslip carrier above the microscope objective with an incubation volume of 300 μL. Cells were covered with either pure Locke's solution or Locke's solution containing the pre-incubation probes as listed above. After incubating the cells with 5 nM Alexa-NA for 15 min, the confocal volume element was positioned to the upper plasma membrane of a cell by motor aided cell scanning in z-direction. At position of half maximal fluorescence of the upper plasma membrane, simultaneous detection of fast diffusing free molecules and slow diffusing ligand bound to the receptor was possible. All measurements were performed for 60 s at 20 °C and cells were used for at best 60 min.

### 5.11 cAMP measurements

cAMP measurements were performed in white 384-well plates coated with 0.1 mg/ml PDL for 30 min and subsequent washing with PBS. 3000 HEK293 GloSensor™ cells were seeded per well and allowed to attach for 16 h. Pre-incubations with α-hederin and the herbal medicinal products were carried out over-night with the following amounts: 1 μM α-hederin, 2.44–9.74 μl/ml ivy preparation, 2.14–8.55 μl/ml ivy/thyme preparation, 0.72–2.89 μl/ml thyme preparation. The highest concentrations of ivy preparation and ivy/thyme combination were chosen to achieve an assay concentration of 1 μM α-hederin. The concentration of the thyme preparation corresponds to the amount of thyme fluid extract present in the ivy/thyme combination. After pre-incubation the medium was changed to substrate solution containing 4 % ATP and 4 % luciferin in HEPES buffered DMEM. The cells were incubated for one hour at 37 °C and subsequently equilibrated for another hour at 24 °C in the plate reader (Tecan infinite 200 Pro). Stimulation was done with 1 μM isoprenaline and 1μM forskolin simultaneously and cAMP increase was measured as heightened luminescence 20 min after stimulation.

### 5.12 Single particle tracking (SPT)

HEK293-β_2_AR-SNAP cells were incubated with 2.89 μl/ml thyme preparation (corresponds to the amount of thyme fluid extract that is present in the ivy/thyme combination), 9.74 μl/ml ivy preparation or 8.55 μl/ml ivy/thyme combination for 12 h. Subsequently, SNAPtag moiety was labeled using 10 nM BG-CF640R in phenol red free DMEM medium for 10 min at 37 °C. Unbound dye was removed by washing with PBS three times. The coverslip was mounted on a coverslip carrier, overlaid with PBS and treated with 10 μM isoprenaline for 20 min. Stimulated cells as well as non-treated control cells were imaged at 20 °C using an EMCCD camera (iXon DV-860DCS-BV, Andor Technology), featuring 128 × 128 pixels of size 24 × 24 μm^2^. The camera is part of a custom-built setup, which uses an inverted wide-field epifluorescence microscope (TE2000-S, Nikon) equipped with a water immersion objective (Plan APO VC, 60x, 1.2 NA, Nikon) and a 200-mm-focal length tube lens. Combined with 4-fold magnification lens (VM Lens C-4x, Nikon), the setup has an effective 240-fold magnification, translating to an image pixel width of 100 nm. A continuous wave laser (Coherent, 637 nm, 50 mW) was used for excitation of labeled receptors. Laser intensity was regulated using an acousto-optic tunable filter (AA Opto-Electronic) and set to 0.7 kW/cm^2^ in the object plane. Cells were imaged in phosphate buffered saline. In order to avoid photobleaching of emitted fluorescence before image acquisition, cells were searched and focused to the epical membrane, before opening the laser shutter and starting the recording. Image sequences were acquired with 20 fps (frames per second). Automated spot detection and track creation were executed by the Track-Mate plugin featured in Fiji software package (https://fiji.sc/). Further computing of diffusion coefficients, their distributions and intratrack transitioning probabilities was done by a Matlab based variational Bayes analysis (vbSPT), which detects diffusion states and can also differentiate between multiple diffusion regimes within one trajectory [14]. Although the vbSPT algorithm is capable to automatically calculate the optimal number of receptor diffusion states, we used a limit of three diffusion states, which led to distinct diffusion coefficients and a better comparability of our data sets. Twenty cells, each recorded with 1000 frames, was found to be a sufficient data set. Jump distance analysis was performed according to the method developed by Anderson et al. (1992) and Smith et al. (1999) [15,16]. This method is based on the probability distribution of jump distances as a function of the lag time. p(r,t)dr is the probability that a particle starting at the origin will be found in a circular ring with the radius r and the width dr after a time t. Different diffusion coefficients $D_{j}$ of different mobility populations were calculated with the following Eq. (4) where M is the total number of jumps and f is the fraction of each population:

(4) p(r,t)dr=∑j=1nMfj2Djtexp(−r24Djt)rdr

### 5.13 Internalization of β_2_AR

HEK293-HiBit-β_2_AR cells were seeded in 96 well plates in a density of 2500 cells per well and allowed to grow another three days to a confluency of ~40–50 % in full-growth medium. Before incubation, the medium was changed to serum-free medium. Cells were incubated with 9.74 μl/ml of the ivy preparation, 8.55 μl/ml of the ivy/thyme combination, and 2.89 μl/ml of the thyme preparation for 16 h. Half of the cells were then incubated with 5 μM isoprenaline for 30 min. The luminescence of HiBiT-β_2_AR at the cell surface was determined using a Tecan Infinite 200 Pro microplate reader and the Nano-Glo® HiBiT Extracellular Detection System (Promega, Mannheim, Germany) according to the manufacturers' instructions. Briefly, the HiBiT tag consists of a small part of the luciferase NanoLuc, which is N-terminally coupled to the β_2_AR. After addition of the LargeBiT (LgBiT), the remaining part of the NanoLuc, a binding to the HiBiT moiety takes place, resulting in a active NanoLuc, which converts added furimazine to furimamide to generate chemiluminescence. The amount of receptors that remained on the cell surface after isoprenaline stimulation and internalization was calculated from the first ten luminescence measurements over a period of 20 min as follows (equation 5):

(5) $$RLU\left(normalized\right)=\frac{\frac{RLU\left(test\;item\;stimulated\right)}{mean\left[RLU\left(test\;item\;unstimulated\right)\right]}}{mean\left\{\frac{RLU\left(control\;stimulated\right)}{mean[RLU\left(control\;unstimulated\right)]}\right\}}$$

### 5.14 Statistical data evaluation

All data points from FCS experiments, cAMP determinations, and β_2_AR internalization investigations represent mean values and standard deviations. The statistical significance of results was proven with one factorial analysis of variance (ANOVA). The results were considered to be significant for p-values ≤ 0.05. Results from SPT experiments are presented as mean values and standard deviations. For statistical analysis the unpaired t-test was performed. Results significantly different from their corresponding control groups are marked by ∗p < 0.05.

## Declarations

### Author contribution statement

H. Bussmann, J. Schulte-Michels, M. Bingel, F. Meurer, S. Aataz and F. Häberlein: Performed the experiments; Analyzed and interpreted the data.

S. Franken and H. Häberlein: Conceived and designed the experiments; Wrote the paper.

### Funding statement

This work was supported by Engelhard Arzneimittel, Niederdorfelden, Germany.

### Competing interest statement

The authors declare the following conflict of interests: Sebastian Franken is an employee of Engelhard Arzneimittel, Niederdorfelden, Germany.

### Additional information

No additional information is available for this paper.
